# An Improved Procedure for the Quality Control of Sintered Carbide Tips for Mining Applications, Based on Quantitative Image Analysis of the Microstructure

**DOI:** 10.3390/ma14051236

**Published:** 2021-03-05

**Authors:** Kamil Mucha, Joanna Augustyn-Nadzieja, Agnieszka Szczotok, Krzysztof Krauze

**Affiliations:** 1 Department of Machinery Engineering and Transport, Faculty of Mechanical Engineering and Robotics, AGH University of Science and Technology, 30-059 Kraków, Poland; krauze@agh.edu.pl; 2 Department of Physical and Powder Metallurgy, Faculty of Metals Engineering and Industrial Computer Science, AGH University of Science and Technology, 30-059 Kraków, Poland; 3 Department of Advanced Materials and Technologies, Faculty of Materials Engineering, Silesian University of Technology, 40-019 Katowice, Poland; agnieszka.szczotok@polsl.pl

**Keywords:** mining, conical picks, WC-Co, sintered carbides, hardness, microstructure, semi-automated measurement, quantitative image analysis

## Abstract

Conical picks are tools that treat surfaces used in mining. Due to a wide variety of such tools, they need to characterise in diversified geometrical and material parameters, as well as durability. For the mining sector, evaluation of the conical picks is performed in order to verify their agreement with the manufacturer’s declaration, as well as with the requirements of the user. One of the whole research procedure elements is a qualitative and quantitative analysis of a pick tip made of sintered carbide (tungsten carbide–cobalt; WC-Co). It is currently carried out when the recipient is accepting such picks or when a complaint is made. In this case, it is important to propose such a research and control procedure that will make it possible to determine the quality of the applied material for pick tips in a straightforward, repeatable, and objective way. This article presents the results of investigations made on tips coming from four different types of conical picks. The tips’ hardness and density were measured, as well as WC grains which were characterised using two kinds of measurements (automated linear-intercept method and semi-automated planimetric methods). The measurements of the WC grains were conducted on microstructure images recorded using a light microscope. The performed investigations made it possible to compare tips and refer the results to those of other scientists’ studies. The final effect is a proposal of a specialised procedure examining picks, especially their tips, made of sintered carbides. This is important for the evaluation of the tips’ quality in light of the requirements of the relevant norms and the needs of the users from the mining and extractive sectors.

## 1. Introduction

During mining, the conical pick is an element which is in direct contact with the exploited undisturbed soil. The picks are mounted in holders fixed on the cutting head, which is part of the mining machine. The picks’ proper operation guarantees its high durability and affects cutting heads’ durability with low energy consumption in the process, as well as low dustiness and scintillation. This is significant for safety reasons. However, obtaining such a result requires appropriately selected kinematic and geometrical parameters of the head together with the whole mining machine as well as geometrical and material parameters of the picks together with the holders [1,2]. To reach the assumed aim, we should ensure an agreement of the mentioned parameters with those assumed at the design stage to obtain the highest possible product quality. By the quality of the picks, we should understand fulfilling the set requirements and achieving the highest possible durability. The acceptable quality of the picks is in the interest of both the users and the manufacturers, because tool replacement, beside the costs related to purchasing and logistics, causes machine shutdowns as well as a drop in efficiency, which means a reduction in the daily output [3,4,5,6,7].

**Figure 1:**
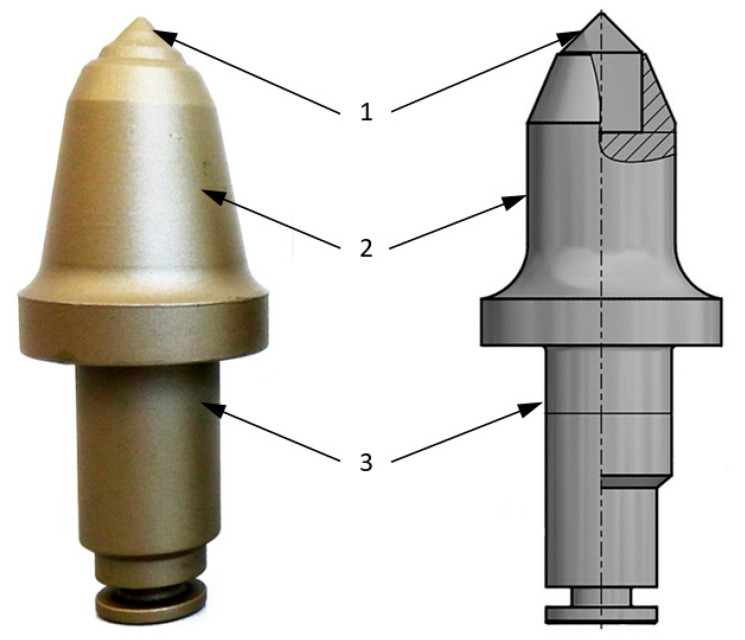
Construction of a conical pick: 1—tip, 2—working part, 3—holder part [4].

A standard conical pick has the shape of a solid revolution consisting of the working part, a one- or two-step cylindrical mandrel, which is called the holder part of the pick, and a tip in the form of an insert made of sintered carbide [1,2,3,4,5,6,7,8] (Figure 1).

The body (the working part and the holder part) constitutes one element made of steel, characterising the appropriate impact resistance, tensile strength, and abrasion resistance. It is recommended that the working part reaches at least 45 HRC, whereas the holder part’s hardness, due to the risk of holder damage, is within the scope of 30 ± 5 HRC. Depending on the tools’ assignment, they are made of different steels, such as 15NiCr13, 41Cr4, 42CrMo4, 36CrNiMo4 or 35HGS. Additionally, the picks undergo thermal and chemical treatment to improve the abrasion surfaces’ resistance most exposed to tribological wear. Alternatively, the surface layer of the functional elements is reinforced with coatings made of stellites or carbides sintered on a base of cobalt, nickel, or iron, for which is used plasma or laser hardening or TIG (Tungsten Inert Gas) or micro-TIG methods [3,4,8]. The preparation of the coatings consists of the use of electrodes in the form of powders or bars. The hardness of the coatings can reach a level of 60 HRC [3,4].

The tip is soldered to the seat in the working part of the picks’ body. It is made of different types of sintered carbides characterising high hardness and reaching its minimum of HV30 ≈ 1050. Most commonly, the applied tips belong to the group of the B grade, i.e., with the volume fraction (Vv) equalling 89–93% of hard abrasion-resistant tungsten carbide, whereas the remaining part is constituted by cobalt, which is the material’s substrate and constitutes the binding phase. The materials usually used for tips of the picks in question are carbides type B1, B2, B20, B23, G15 and other materials with similar chemical compositions and properties [3,4,5,7,8].

This article analyses the quality of the tips made of sintered tungsten carbide–cobalt (WC-Co), a material characterising high abrasion resistance [9], hardness, and impact strength. It is less brittle than stellites, corrosion-resistant, and maintains its properties even up to 850 °C [4,10].

Sintered metals are a range of very hard, refractory, wear-resistant alloys made by powder metallurgy techniques [11]. The beginnings of sintered carbides’ production date back to 1923, when they were developed and patented. In 1926, their industrial application was initiated [12]. The global production of sintered carbides has grown in the past 30 years [13]. Almost 65% of the total sintered carbide production is related to metal cutting tools. Mining, oil drilling, and rock industries hold about 15% of the market share, whereas wood and construction industries account for 10%. Compared to other hard materials, sintered carbides make up 50% of the total world market; high-speed steels are 45%, ceramics about 4%, and polycrystalline diamond (PCD) and cubic boron nitride (CBN) 1% [14]. In the tool industry, sintered WC-Co is one of the most critical materials. They are applied to construct such tools as milling machines, burrs, and multi-blade cutting plates [15,16,17]. Sintered WC-Co is commonly used in the mining and construction industry [1,2,3,4,5,6,7,8,18], as well as agriculture [19]. The widespread use of cemented carbides such as tungsten carbide–cobalt (WC-Co) as cutting tool materials is primarily due to their unique combination of desirable properties such as high hardness, strength, resistance to compressive deformation, and wear resistance [20].

According to the data presented in Przybyłowicz [21], sintered carbides consist of a cobalt matrix (up to 25%) and various carbides, usually WC, but also TiC, TaC and NbC. As mentioned by Boshcarbide [22], sintered carbides consist mainly of tungsten carbide (WC), which forms the hard phase with a proportion between 70% and 97%; cobalt (Co), which serves as a binder with a proportion between 3% and 27%; and small portions of different carbide, such as titanium carbide (TiC), tantalum carbide (TaC), or niobium carbide (NbC). Based on Kresse et al. [23], the most commonly produced commercial straight grades of WC-Co have cobalt contents ranging from 4 to 30% by weight, with grain sizes ranging from 0.5 to 10 μm. In work by Nohak et al. [24], the authors have investigated different commercial WC-Co hard metals with carbide grain sizes ranging from ultrafine to coarse, and Co contents between 4.2 and 25 wt.%. As reported by Mingard et al. [25], the hard phase, i.e., tungsten carbide (WC), constitutes the main part of cemented carbide because it may present up to 70–90% by weight. Based on Liu et al. [26], there are WC-Co compounds even with 30.8 vol.% of Co.

Microstructural studies of sintered carbides are of great significance for understanding their properties and engineering performances. There are many factors that have a direct bearing on the microstructure of sintered carbides. Among others, there is the elemental chemical composition of the hard and binder phase, as well as the shape, size, and distribution of WC particles. One of the most convenient tools has been semi-automatic analysis based on magnified light optical micrographs (LM) of etched specimens. Currently, automatic image analysis using scanning electron microscopy (SEM) is widespread. However, the high magnification used in SEM to image the grain boundary network and the fine carbide grains makes it challenging to obtain a representative result for coarse carbide grains [27].

Investigations on the complexions in the WC-Co cemented carbides are still very limited. Its formation mechanism, stability, and effects on cemented carbides’ and mechanical properties have remained unclear [28].

It was noted that wear rate was inversely dependent on hardness [29]. Wear is the parameter that can be used for the performance evaluation of coal/rock cutting equipment. The tool life of bits is limited by their wear rate [24]. The grain size of carbide affects the wear-resistance property of the cemented carbide. The coarse-grained carbides usually have a lower abrasive wear resistance than a fine or medium grain size [30,31]. The results showed in Wang et al. [31] pointed out that the grain size had opposite effects on wear resistance of the cemented carbides in dry sliding wear and microabrasion tests. Referring to Deng et al. [32], by increasing the granule size of carbides, the fracture toughness increases. According to Pirso et al. [33], Larsen-Basse [34] and Jia et al. [35], the tribological, mechanical and thermal properties of WC-cemented carbide substantially depend on its composition and WC particle size. The grain size of carbide affects the wear-resistance property of cemented carbides. The coarse-grained carbides usually have a lower abrasive wear resistance than a fine or medium grain [30,33].

Brookes [36] has concluded that the bulk composite hardness decreases with an increase in binder content. It was found that for most of the hard metal compositions, at least for straight WC-Co, the measured values of binder phase tally vary with the nominal composition [37]. Transverse rupture strength increases with an increase in cobalt content. The increase in Co may be up to 20%. The further increment of Co leads to the separation of carbide grains [38].

The problem with the application of sintered WC-Co consists mainly of a distinction between the user’s expectations and the offer of the manufacturer; it is necessary to apply the proper research procedure assessing the sintered carbide tips, however not at the production stage but the stage of the ready product. Users (miners) want to ensure that the delivered conical picks comply with their requirements and the manufacturer’s (supplier’s) declarations. WC-Co tips in the conical picks delivered to the user do not have the manufacturer’s parameters. The parameters that are checked when assessing the tip include linear dimensions, HV30 hardness, specific density, chemical composition, the mass fraction of carbide phase (% WC) and matrix (% Co) as well as the size of WC particles. The method of measuring WC grains’ size has been specified in the ISO 4499-1:2020 standard [39]. However, in this method, the human (operator) determines the grain boundary and boundary contour be measured, so the human factor’s effect on the test accuracy is significant. That is why the proposed research method limits the operator’s effect by applying the same macro with image transformation procedures in all the examined pick tips. Macros are handy tools to automatise repetitive tasks standardise and document the image processing efforts [40].

This study’s objective is to present a modified scientific procedure assessing the sintered carbide tips as a recommendation for its use for a ready product.

## 2. Materials and Test Methodology

**Figure 2:**
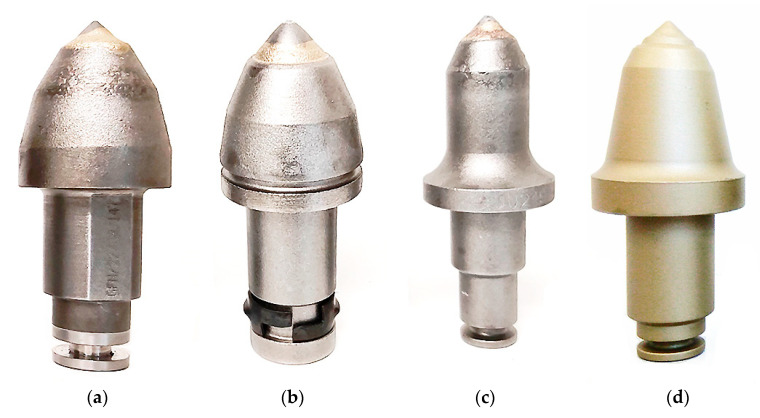
Compilation of picks assigned for test tip collection: (**a**) Road 1, (**b**) Road 2, (**c**) Longwall 1, (**d**) Longwall 2.

The examinations were performed for four different types of conical picks. The examined picks included both those assigned for longwall shearers and roadheaders from different manufacturers. The following picks were selected for the tests (Figure 2):

- Commercial pick for roadheaders—marked as Road 1 (Figure 2a);
- Commercial pick for roadheaders—marked as Road 2 (Figure 2b);
- Commercial pick for longwall shearers—marked as Longwall 1 (Figure 2c);
- Commercial pick for longwall shearers—marked as Longwall 2 (Figure 2d).

**Figure 3:**
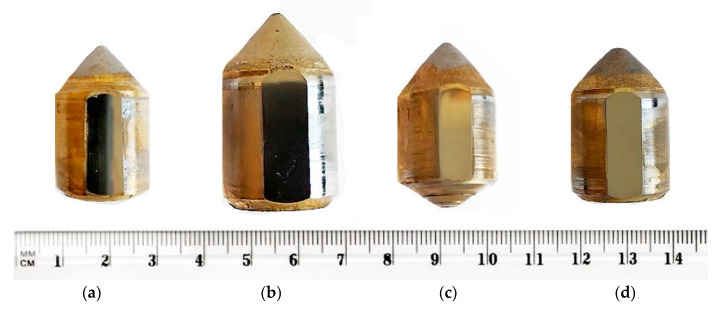
Comparison of sintered carbide tips assigned for the tests: (**a**) Road 1, (**b**) Road 2, (**c**) Longwall 1, (**d**) Longwall 2.

According to the research procedure described in Krauze et al. [5], a measurement of each pick’s linear dimensions was made. Next, the appropriately prepared samples underwent a chemical composition analysis (the pick’s working part) to determine the steel grade and hardness measurements (HRC), microscopic observations, whose aim was a qualitative and quantitative analysis of sintered carbide tips. The tips made of sintered carbide were separated from the steel bodies of the picks (Figure 3) by way of machining (turning) and subjected to geometrical and hardness measurements (HV30) according to PN EN 23878 standard [41]; additionally, their density was evaluated based on ISO 3369 standard [42], and the tungsten (W) and cobalt (Co) carbide contents were determined referring to ISO 4499-1:2020 standard [39] and PN-88/H-89500 standard [43]. Fragments of the four tips made of sintered carbides were selected for quantitative microstructural tests. Sintered carbides are hard, which alters the preparation procedure.

Grinding and polishing processes were conducted according to recommendations presented in ISO 4499-1:2020 [39]. During grinding, material removal to obtain a plane surface with minimal damage was performed using grain sizes of 100, 320, 500, 800, 1000 and 1200 µm. Rough polishing was accomplished primarily with diamond abrasives ranging from 1 μm to 3 μm. Final polishing was carried out with polishing cloth OPS and diamond abrasives 0.25 μm diamond. Grinding and polishing consumables were provided by the Struers company [44]. The prepared microsections underwent chemical etching in a reagent of the following composition: 15 mL H_2_O + 30 mL HCl + 15 mL CH_3_COOH and 15 mL HNO_3_ to reveal the grain boundaries of the W carbides. The sample etching was carried out at the temperature of 20 °C, and the etching time was from 1 to 3 min, depending on the sample.

The measurement of the carbide grains in the cobalt matrix was performed using an image analysis program Met-Ilo [45]. Two kinds of WC grain measurements (automated linear-intercept method and semi-automated planimetric methods) were proposed to determine the parameters describing their size and shape, contiguity in WC-Co, and relative volume in the materials of the tips. Two microphotographs were registered using a light microscope (LEICA DM 4000 M) and a scanning electron microscope (HITACHI S-3500N). The W carbide grain measurements were made on the materials’ microstructure images recorded using a light microscope. The microphotographs recorded with magnification 200× were treated as demonstrative and suitable for comparison, but they were not subjected to a quantitative analysis due to the difficulties in interpreting the WC grain boundaries. For further research, the microphotographs recorded with magnification 500× were selected. The series of microphotographs from the light microscope in every pick was analysed.

The linear-intercept method and the planimetric method were applied for the measurement of the WC grains. In the linear measurement, 50 parallel test lines were applied, the length of which corresponded to the longer dimension of the measurement image plotted onto each measured image. The program marked the chords crossing the analysed carbides on the consecutive lines and measured those chords’ particular lengths.

The authors of this manuscript confirm that the number of chords in one field of view depends on the used magnification and analysed carbides’ dispersion. Referring to Ryś [46], the more significant chords with lower magnification, the lower the measuring accuracy.

For the planimetric method, understanding is vital to notice that every investigated image was just a set of different pixels. Every pixel belongs to a particular class (either background or carbide). Firstly, operations of a 3 × 3 median filter were conducted on greyscale images during the pre-processing. The purpose of the image segmentation here was to separate the image into the background and target areas (carbides). As a result, all the pixels belonging to the carbides were represented by the same colour (carbide as red). The segmentation quality directly affects the feature extraction. In the proposed procedure, the image was segmented by the maximum inter-class variance algorithm. It calculates the inter-class variance (between classes/segments) and the intraclass variance (within a particular segment). The algorithm’s main objective is to obtain the minimum value for the intra-class variance and the maximum value for the inter class variance. It means that the pixel intensities within a segment must be close to each other, while the pixel intensities must be well separated across different segments. After applying the algorithm, a segmented image is obtained, which is composed of many independent regions. The final step was a manual correction in the areas where it was necessary to obtain the final binary image to measure the carbide grains.

In the planimetric method, a measurement of the area of plane W carbide, section (A), visible in the image, was made, and then it was referred to the total area of the image (A_A_—area fraction of the carbides, which is an estimator of volume fraction V_V_). In this measurement, the mean diameter ($\bar{d}$) was also used, which was determined as the mean value out of 36 measurements of the Ferret diameters defined for each carbide at different angles. To evaluate the shape factor of the particles, different indicators were applied. The shape factor of the carbides applied here was determined as the dimensionless ratio between areal and linear parameters describing WC grains [46]:

(1) $$\xi =4\pi \frac{A_{i}}{P_{i}^{2}}$$

where *P_i_* is the perimeter of carbide (μm), and *A_i_* is the plane section area of carbide (μm^2^).

A shape factor value at the level of 1 means a circle. Assuming that the images are representative of the specimen, the volume fraction of the carbides is measured by their area fraction (A_A_), so the area fraction of the carbides (A_A_) is an estimator of their volume fraction (V_V_). Besides the number of carbides, the number of carbides per unit area of the plane section (N_A_) was also evaluated.

The coefficient of variation given with various parameters denotes the heterogeneity index of the given parameter. It is a classic measure of diversification in the distribution of a given characteristic/parameter. Unlike the standard deviation, which describes the parameter’s absolute diversification, the variation coefficient is a relative value, so it depends on the arithmetic mean value. It was calculated as a ratio of the standard deviation to the arithmetic mean from the given parameter measurements. The specific surface area of the carbide boundaries (*S_v_*) is intimately linked to the particle size because it is estimated based on the following formula [46]:

(2) $$S_{v}=\frac{4L_{A}}{\pi }$$

where *L_A_* means the specific length of the carbide boundaries (μm).

The relative specific surface of the carbides is evaluated as *S_v_*/*V_v_*. A higher value of *S_v_*/*V_v_* proves a more significant refinement of the carbide phase.

Contiguity was defined by Gurland in 1957 [47], as “the fraction of the total internal surface area of a phase that is shared by particles of the same phase”. Therefore, by definition, the contiguity can vary between 0 and 1. In WC-Co, the carbide phase’s contiguity has been assumed to tend to zero when the cobalt volume fraction tends to one, and contiguity tends to one when the cobalt volume fraction tends to zero. In work by Mingard et al. [25], the contiguity is described as a measure of the proportion of the carbide grain boundaries that are in direct contact with other carbide grain boundaries. This measure is an important characteristic of the microstructure of hard metals and is likely to play an essential role in determining mechanical, electrical, and thermal properties through its effect on interface structure. According to Exner et al. [48], contiguity effectively forms a skeleton of WC. Many of the unique properties of cemented carbides are due to the formation of the WC skeleton, the occurrence of which in many cases makes a simple rule of mixture predictions invalid for cemented carbides. Contiguity in this work was evaluated according to the following formula [48]:

$$C=0.074{*V}_{\mathrm{Co}}^{-1}$$

where V_Co_ is the volume fraction of the Co binder (%).

## 3. Test Results

The conducted analysis of the steels’ chemical composition from which the bodies of all four picks had been prepared showed that they were made of one steel grade 100CrMn6, cold work tool steels. The hardness examined picks’ working part was within the scope of 35–44 HRC, and the holder parts 38–41 HRC.

Each of the four picks assigned for the tests contained a conical–cylindrical tip. The tip diameter in the case of three tips (Road 1 and Longwall 1 and 2) equalled 22.0 mm, while in the case of the tip of Road 2, it was 25.0 mm. The length of all the tips equalled 35.0 ± 0.1 mm. These parameters’ measurements were made on a marking-off table using a slide calliper with an accuracy of up to 0.01 mm.

Table 1 presents the results of the hardness (HV30) and density measurements performed on the examined picks’ tips. Based on these results, according to PN-88/H-89500 standard [43], the tip’s carbide type was determined.

**Figure 4:**
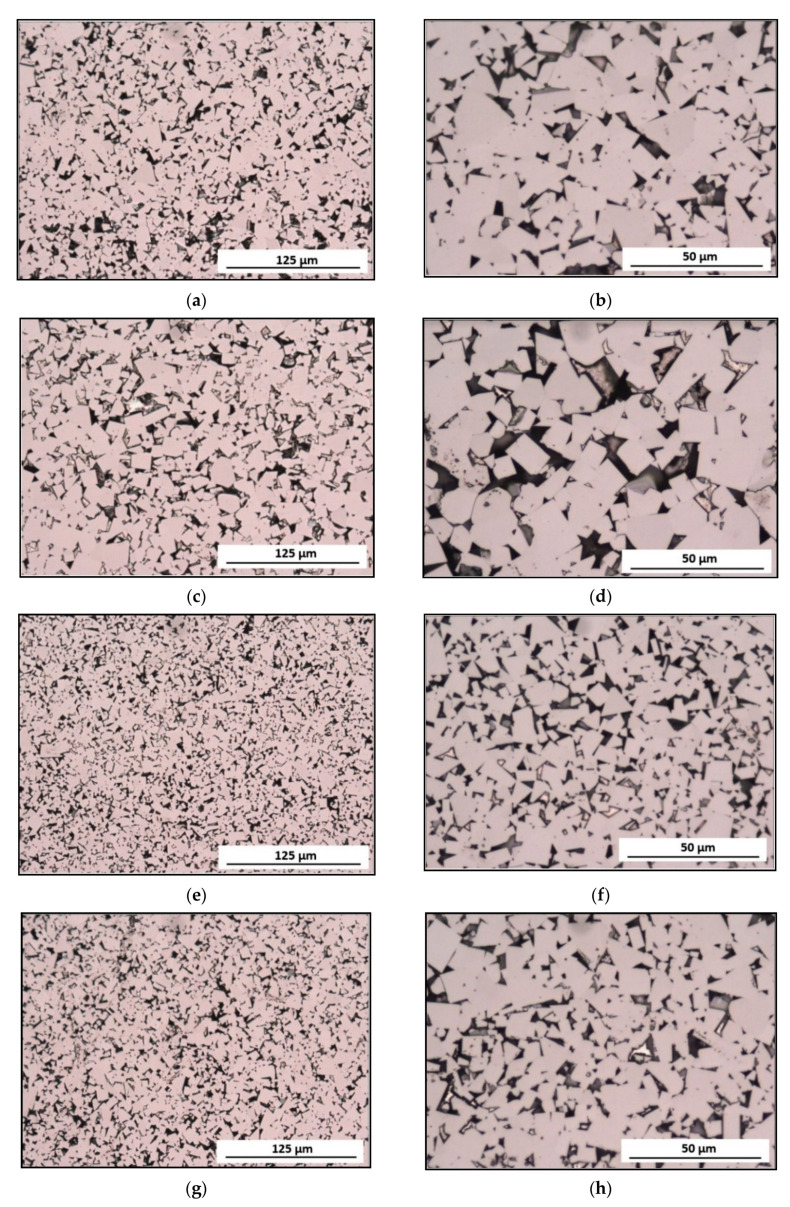
Microstructures of the examined tips made of sintered tungsten carbide–cobalt (WC-Co): (**a**,**b**) Longwall 1 tip, (**c**,**d**) Road 1 tip, (**e**,**f**) Longwall 2 tip, (**g**,**h**) Road 2 tip. Light Microscope (LM), Bright Field (BF).

Figure 4 compiles exemplary microstructures of the tip materials of the examined picks. Figure 4a,c,e,g show the test materials’ microstructures with lower magnification (200×), and Figure 4b,d,f,h illustrate these microstructures with a higher magnification (500×).

The microscopic test results revealed differences in the amount and refinement of the carbide phase (WC) in the matrix Co. The carbide grains had a multilateral splinter shape in each of the examined tips on sharp edges and flat surfaces (Figure 4a–h). In the tips of Longwall 2 and Road 2 (Figure 4e–h), refinement of the WC grains was observed compared to the tips of Longwall 1 and Road 1.

**Figure 5:**
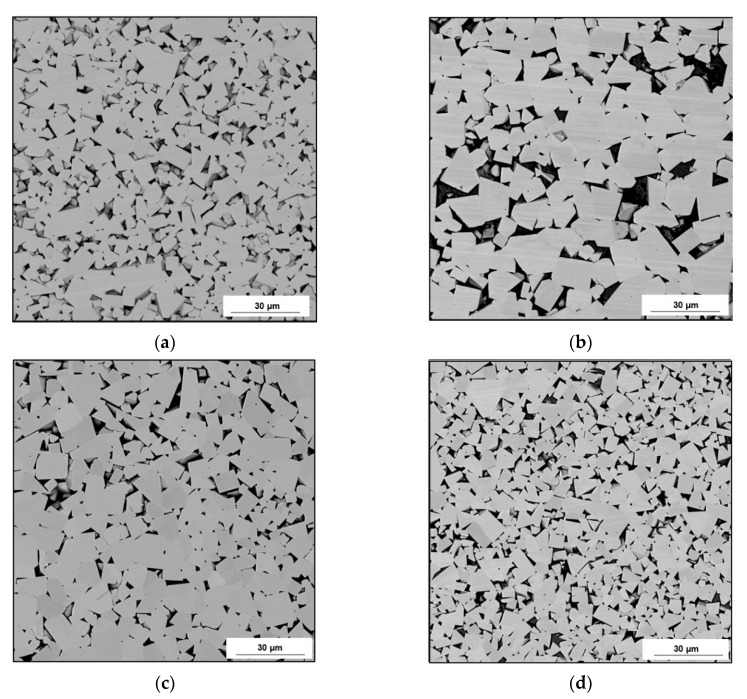
Microstructures of the examined tips made of sintered WC-Co: (**a**) Longwall 1 tip, (**b**) Road 1 tip, (**c**) Longwall 2 tip, (**d**) Road 2 tip. Scanning Electron Microscope (SEM), Back Scattered Electron (BSE).

The same samples were observed using SEM, and the selected microphotographs are presented in Figure 5.

**Figure 6:**
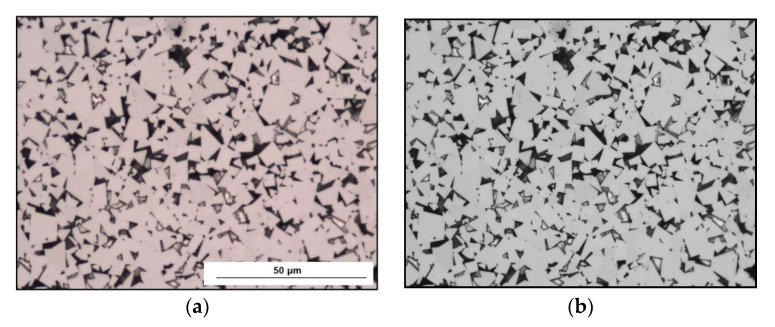

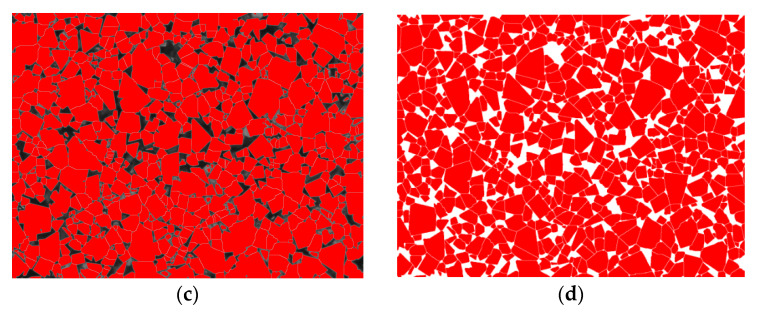
Compilation of the crucial image conversions for WC grain abstract: (**a**) initial image, (**b**) median filter, (**c**) segmentation, (**d**) binary image of carbides after manual correction.

A set of essential steps of the image analysis procedure for planimetric measurement of the carbides is presented in Figure 6.

The discussed parameters characterising the described sintered carbide grains obtained from a whole series of measurements in the examined materials have been compiled in Table 2.

**Figure 7:**
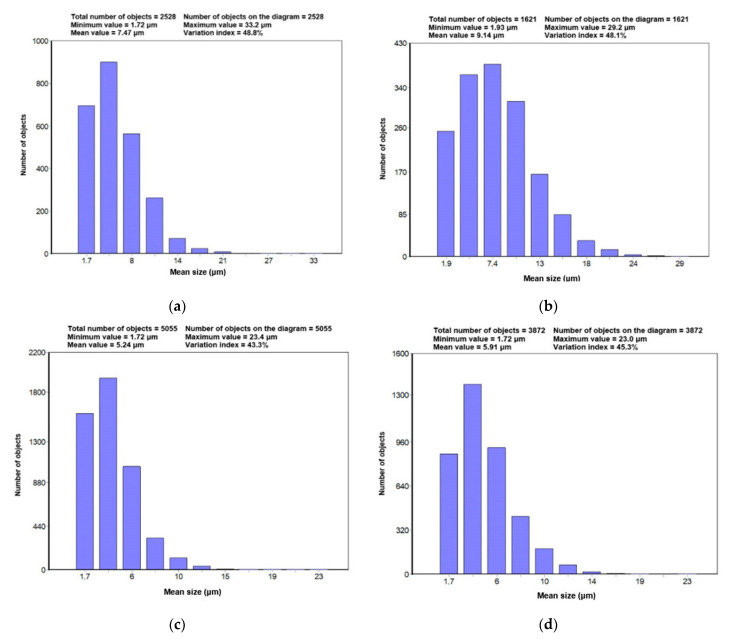
Diagrams of statistical distributions of mean carbide grains sizes in particular tips: (**a**) Longwall 1 tip, (**b**) Road 1 tip, (**c**) Longwall 2 tip, (**d**) Road 2 tip.

**Figure 8:**
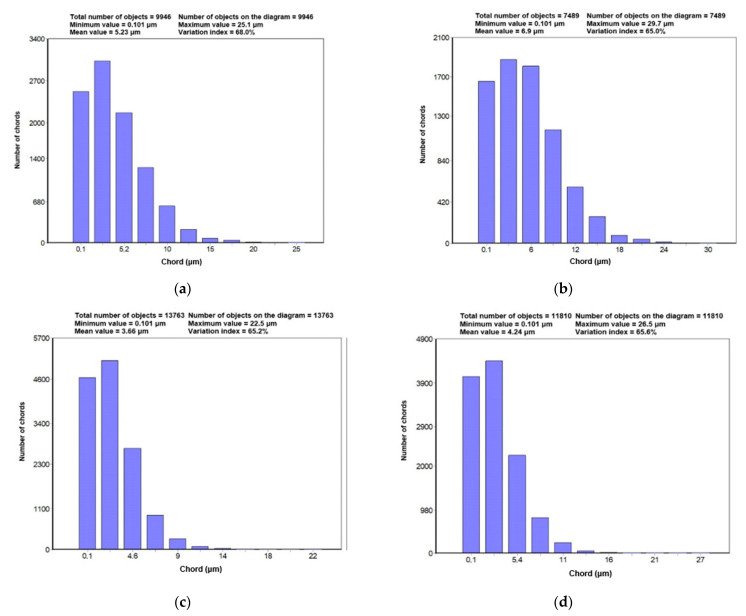
Diagrams of statistical distributions of chord lengths intersecting WC grains in particular tips: (**a**) Longwall 1 tip, (**b**) Road 1 tip, (**c**) Longwall 2 tip, (**d**) Road 2 tip.

Based on the performed measurements, diagrams of the statistical distributions of the WC grains’ mean diameter in the particular tips were created, shown in Figure 7. Additionally, Figure 8 presents the diagrams of statistical distributions of the chord lengths intersecting the WC grains.

The performed measurements of the carbide grains in the tips of tangential-rotary picks provided the following results: the smallest carbides and their highest number was recorded in the tip of Longwall 2, whereas slightly larger carbides with a reduced number were present in Road 2, and even bigger carbides with even further reduced numbers were observed in Longwall 1, and the biggest carbides with the smallest number were recorded in Road 1. The carbide size gradation was confirmed by the results of both the linear measurement (length and number of chords) and the planimetric measurement (mean carbide diameter, the surface area of plane carbide section). It is visually verified in the microphotographs shown in Figure 4 and Figure 5.

The measurement of the mean size of the analysed WC grains ($\bar{d}$) in the particular tips of the picks has provided the following information: the scope of the value of mean WC grain size equalled 5.24 μm (Longwall 2) to 9.14 μm (Road 1). In the tips of Road 2 and Longwall 1, the mean size of the WC grain assumed intermediate values, i.e., 5.91 μm and 7.47 μm.

Comparing the mean values of the WC grain plane section area in the particular tips confirmed that the value scope is from 19.42 μm^2^ (Longwall 2) to 62.13 μm^2^ (Road 1). In the tips of Road 2 and Longwall 1, similarly to the case of the WC’s value, the values of the WC plane section area were intermediate, i.e., 25.15 μm^2^ and 40.05 μm^2^, respectively.

The refinement of the WC grains can also be concluded from the parameter S_v_/V_v_ (Table 2). This parameter’s highest value is in Longwall 2, where the highest number of carbides was recorded, and the carbide grains were the smallest of all four analysed pick tips. The lowest value of this parameter was in the case of Road 1, where the lowest amount of carbide grains and the biggest size were observed.

The WC grains’ shape factor was similar in all the examined pick tips; it reached its mean value from 0.74 to 0.77 and was characterised by a relatively low coefficient of variation (from 13.15% to 17.32%).

The presented measurements confirmed that the WC phase’s area fraction of the WC phase equalled: 80.76%; 80.33%; 78.31%; and 77.67% in all four examined tips, respectively. This parameter’s value could be a little underestimated because, at the stage of selecting the carbide grains to be assigned for the measurements, the microstructures with a surface area below 12 pixels were eliminated as artefacts. This is related to the fact that digital images are often affected and corrupted by various noise and artefact types. The sources of artefacts could be the instrumentation used or the environment of the experiment. The removal of artefacts without causing any distortion or loss of the desired information in the image of interest is often a significant challenge [49].

In the comparison of the area fraction of WC (V_v_) with the mean carbide diameter, the length of the chords crossing the carbide and the area of a plane carbide section, it can be observed that, in the case of all those parameters, smaller values were obtained for the tips of Longwall 2 and Road 2 than in the case of Longwall 1 and Road 1.

## 4. Discussion

The tips of machining picks are made of sintered carbides. Most commonly, tips with a conical–cylindrical shape and diameters of 22 mm and 25 mm are applied. Based on the performed investigations, it has been established that the highest hardness was exhibited by the tips of Longwall 1 and Road 1, i.e., 1152 HV30 and 1146 HV30, respectively. Based on the microstructural observations and hardness measurements, a relationship between this property and the area fraction (V_V_) of the WC grains can be stated, which classifies itself as follows: 80.76% WC for the tip of Longwall 1; 80.33% WC for the tip of Road 1; 78.31% WC for the tip of Longwall 2; and 77.67% WC for the tip of Road 2.

According to the classification of WC-Co microstructures for different WC grain sizes presented in Garcia et al. [13], based on Ortner et al. [50] the results of the carbides measurements obtained in the present research indicated that these carbides are in an extra coarse class (size > 5 μm).

The grain size of carbide phases is the most carefully controlled variable besides carbon control in the production of sintered carbides. The obtained results of WC grain sizes confirm the results presented in the work by Garcia et al. [13] concerning the application of WC with grain sizes between 2 μm and 10 μm in the resource extraction area.

The results of the performed measurements of the hardness of cemented carbides (above 1100 HV30), WC grain size and cobalt content (vol.%) also comply with data worked out by Upadhyaya [51] and presented in Yang et al. [52], i.e., with approximately 20 vol.% of Co, grain size between 5 and 10 μm, and hardness between 1100 and 1200 HV.

One can find a range of contiguity values in the literature with the information about the cobalt volume fraction in selected cemented carbide. This work’s experimental results indicate that contiguity results with values from 0.33 up to 0.38 are conformable with those presented in Mingard et al. [25].

Based on the microstructural observations of the examined tips, we can establish that the microstructure consists of grains of W carbides and a cobalt matrix, which plays a binder’s role. It is well known that a crucial role is played by the size of the WC grains and the cobalt matrix’s content. In the performed investigations, it has been demonstrated that the cobalt content (vol.%) was relatively high (approximately 20%) and in the investigated tips, the carbide grains had a multilateral splinter structure, with sharp edges and flat surfaces.

The parameters describing the WC’s grains’ size and shape made it possible to compare the WC grains in all the examined pick tips. Table 2 provides the possibility to compare the averaged results of these measurements, while Figure 7 and Figure 8 show the measured minimal and maximal values of the analysed parameters in the particular pick tips.

It is common knowledge that the WC grains’ size is one of the most important parameters used to adjust the relationship between the hardness and impact strength of the given grade, which was mentioned in the literature review. According to the data included in Garcia et al. [13], more refined grains mean higher hardness with the given content of the binder phase. The amount and composition of a cobalt-rich binder make it possible to control the grade’s impact strength and its plastic deformation resistance. With the same size of the WC grains, increasing the binder amount will increase the grade’s impact strength, which will be sensitive to wear caused by plastic deformation. An insufficient amount of the binder may make the material brittle. According to European Hard Materials Group [14], this is important because a volume fraction of SC (V_v_) above 80% is crucial for the application of WC-Co in machine cutting.

The full automatization of WC grain measurement in the linear method and part-automatic planimetric method using sequences of filters and transformations made it possible to introduce repeatable carbide segmentation conditions on all the examined samples. The operator–analyser’s involvement was limited to verifying the segmentation results’ properness and correcting the errors before the carbide measurement.

It was also critical to determine the initial measurement conditions, including the determination of the minimal values considered for the objects’ measurements to eliminate minor artefacts visible in the microphotographs [49].

## 5. Conclusions

The presented methodologies of determining the carbide size in the examined tips (linear and planimetric) complement each other. The planimetric method, which provides two- and three-dimensional image parameters characterising the carbides, can be applied as a verifying tool of the carbide grain size obtained by the linear method and complementing the carbide grain descriptions. In this way, the determination of the tip quality, which has so far been problematic, especially in the size of the WC grains, becomes more accurate. This is why the discussed research procedure is recommended for practical use in evaluating tips made of sintered carbides (WC-Co) at the stage of submitting an offer or a complaint.

The obtained parameters describing the studied material comply with others presented in the literature.

## Figures and Tables

**Table 1:** Comparison results of the sintered carbide tips.

No.	Tip Denotation	Hardness, HV30	Density, g/cm^3^	Carbide Type
1	Longwall 1	1152 ± 29	14.621	B23
2	Road 1	1146 ± 22	14.572	B23
3	Longwall 2	1128 ± 28	14.483	B40
4	Road 2	1138 ± 27	14.378	B40

**Table 2:** Measurement results for the particular parameters of carbides in the examined tips.

Parameter		Longwall 1	Road 1	Longwall 2	Road 2
Number of measured carbide grains	-	2528	1621	5055	3872
Number of carbide grains per unit area of plane section N_A_	(1/mm^2^)	20,162.86	12,928.79	40,317.74	30,882.35
Length of chords (d_WC_)	Mean (μm)	5.23	6.90	3.66	4.24
	Standard deviation (μm)	3.56	4.49	2.39	2.78
	Coefficient of variation (%)	68.02	65.03	65.22	65.61
Number of chords	Mean	994.6	748.9	1376.00	1181.00
	Standard deviation	57.43	26.47	31.87	51.70
	Coefficient of variation (%)	5.48	3.35	2.20	4.15
Mean size of carbide grains ($\bar{d}$)	Mean (μm)	7.47	9.14	5.24	5.91
	Standard deviation (μm)	3.65	4.40	2.27	2.68
	Coefficient of variation (%)	48.79	48.09	43.34	45.27
	Limiting error δ_gr_ of mean WC grains size * (μm)	0.14	0.21	0.06	0.08
Area of plane carbide grain section (A)	Mean (μm^2^)	40.05	62.13	19.42	25.15
	Standard deviation (μm^2^)	41.33	62.43	18.88	25.23
	Coefficient of variation (%)	103.2	100.5	97.22	100.3
Shape factor of carbide grains	Mean	0.74	0.74	0.77	0.76
	Coefficient of variation (%)	17.32	16.71	13.15	14.89
Area fraction of carbides (A_A_) which is an estimator of volume fraction (V_V_)	Mean (%)	80.76	80.33	78.31	77.67
	Standard deviation (%)	2.27	2.89	1.82	1.25
	Coefficient of variation (%)	2.67	3.41	2.20	1.53
	Absolute error of WC Vv with assumed significance level 0.05 (%)	4.52	5.54	3.01	3.47
Relative specific surface of carbide grains (Sv/Vv)	(mm^2^/mm^3^)	759.49	594.87	1084.05	951.64
Contiguity (Cwc/wc)	-	0.38	0.38	0.34	0.33

* The limiting errors δ_gr_ of the mean WC grains’ diameter in the particular tips were estimated according to the formula: $\delta_{\mathrm{gr}}=u_{\alpha }\frac{s}{\sqrt{n}}$ with the assumed significance level α = 0.05, for which u_α_ = 1.96, where s is the standard deviation from the mean grain diameter measurements.

## Data Availability

Data presented in the article are original and not inappropriately selected, manipulated, enhanced, or fabricated.
